# Photoplethysmography Feature Extraction for Non-Invasive Glucose Estimation by Means of MFCC and Machine Learning Techniques †

**DOI:** 10.3390/bios15070408

**Published:** 2025-06-24

**Authors:** Christian Salamea-Palacios, Melissa Montalvo-López, Raquel Orellana-Peralta, Javier Viñanzaca-Figueroa

**Affiliations:** 1 Interaction, Robotics and Automation Research Group, Salesian Polytechnic University, Cuenca 010102, Ecuador; mmontalvol@ups.edu.ec (M.M.-L.); fvinanzaca@est.ups.edu.ec (J.V.-F.); 2 Genetic Improvement and Global Production in Livestock Species Research Group, Salesian Polytechnic University, Cuenca 010102, Ecuador; sorellanap@ups.edu.ec

**Keywords:** biomedical systems, medical applications, signal analysis, processing techniques, neural networks, regression algorithm systems

## Abstract

Diabetes Mellitus is considered one of the most widespread diseases in the world. Traditional glucose monitoring devices carry discomfort and risks associated with the frequent extraction of blood from users. The present article proposes a noninvasive glucose estimation system based on the application of Mel Frequency Cepstral Coefficients (MFCCs) for the characterization of photoplethysmographic signals (PPG). Two variants of the MFCC feature extraction methods are evaluated along with three machine learning techniques for the development of an effective regression function for the estimation of glucose concentration. A comparison between the performance of the algorithms revealed that the best combination achieved a mean absolute error of 9.85 mg/dL and a correlation of 0.94 between the estimated concentration and the real glucose values. Similarly, 99.53% of the validation samples were distributed within zones A and B of the Clarke Error Grid Analysis. The proposed system achieves levels of correlation comparable to analogous technologies that require earlier calibration for its operation, which indicates a strong potential for the future use of the algorithm as an alternative to invasive monitoring devices.

## 1. Introduction

It is estimated that 451 million people worldwide are currently diagnosed with Diabetes Mellitus [1]. Patients are advised to monitor their glucose levels in order to prevent extreme glycemic episodes and avert complications related to the disease such as diabetic retinopathy, ulceration, stroke, and chronic kidney failure [2]. Traditional self-glucose measurement requires blood to be applied to a glucometer’s test-strip. Drawing blood from the body leads to pain and discomfort in patients, and it is known to carry inherent risks of wound infection and fluid transmitted diseases, as well as other disadvantages associated with the expenses of disposable needles and strips.

Non-invasive glucose measurement techniques have been investigated for the past three decades, as they offer a promising alternative to the drawbacks associated with invasive systems. The developed non-invasive technologies are generally classified into optical, thermal and transdermal methods, with near-infrared spectroscopy and reverse iontophoresis being the most extensively studied. Although some studies have reported favorable results, only a limited number of commercial devices are currently available to the public [3]. In order to achieve a reliable and accurate self-monitoring method, further research and extensive testing are still required, particularly in areas of sensor development and raw data processing.

Indirect glucose measurement is a promising technique in which glucose levels are estimated based on the existing correlation between glucose concentration and its effects on the physical properties of blood, capillaries and tissues [4]. In contrast, direct measurement methods often face limitations inherent to the molecular characteristics of glucose. Glucose molecules are colorless, smaller than hemoglobin, and unevenly distributed throughout the body [5], factors that collectively contribute to the ongoing challenge of accurate glucose measurement.

Glucose has a direct impact on blood osmolarity. Studies have demonstrated that blood viscosity is strongly associated with glucose concentration and blood pressure [6,7]. This effect is particularly evident in hyperglycemic states, where a reduction in plasma volume leads to an increase in hematocrit levels. Furthermore, in [8] it is suggested that overall blood viscosity varies proportionally with the degree of hyperglycemia in patients with diabetes and prediabetes.

Photoplethysmography (PPG) is a technique used to measure changes in blood volume within the body [9]. A PPG device consists of a light emitter that illuminates the tissue and a photodetector that senses the reflected light. The amount of light absorbed varies in response to fluctuations in blood volume within the circulatory system, producing a PPG signal that contains information related to the autonomic, respiratory and circulatory systems [10]. Processing this signal may reveal new insights into the hemodynamic characteristics of the body, as well as specific blood components [11].

PPG signals can serve as a valuable medium for detecting the relationship between vascular behavior and glucose concentration, as variations in blood flow and viscosity are reflected as changes in the waveform in both time- and frequency-domain analyses. The analysis of the signal spectrum can identify intrinsic patterns of the function, as well as information about the source and propagation medium.

Human body signals typically contain information from multiple physiological systems and processes. To isolate the target biological behavior from the entire physiological signal, methods capable of transforming raw signals into meaningful features must be applied. In the case of voice signal analysis, the Mel-Frequency Cepstral Coefficient (MFCC) technique is able to model the dynamics of the vocal tract as a speech modulation filter, where the biological characteristics become additive components of the waveform in both the cepstral and frequency domains [12]. The behavior of the circulatory system can be characterized using this same technique applied to the signal of change of blood volume PPG and its harmonics, which represent the intrinsic hemodynamic characteristics of the circulatory system.

**Figure 1:**
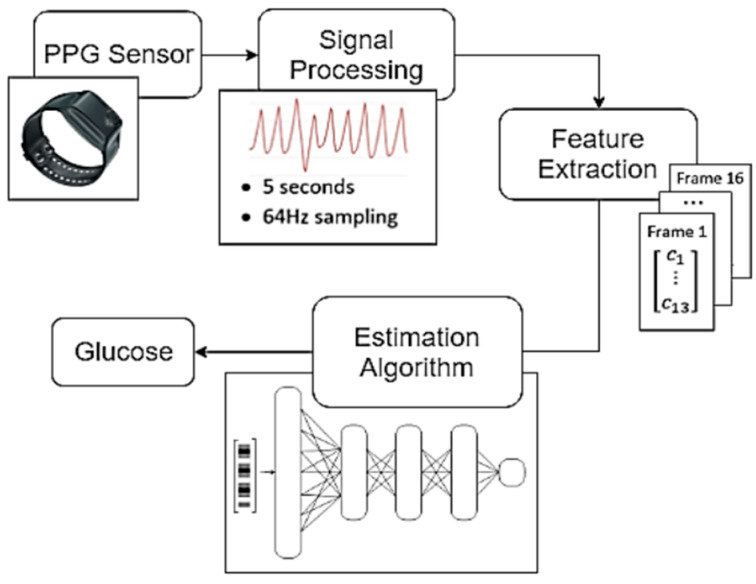
Structure of the proposed system. The main blocks are Signal Processing, Feature Extraction, and Estimation Algorithm.

The present article proposes a glucose estimation system based on the correlation between glucose and blood viscosity, as reflected in the body’s PPG signals and characterized through MFCC features. The proposed glucose estimation system consists of three main analytical modules (see Figure 1). The first module refers to the Signal Processing techniques used to distinguish corrupted raw PPG signals and organize the valid ones for further analysis. The Feature Extraction module introduces three MFCC-based approaches for signal characterization aimed at effectively representing hemodynamic information in each case. These approaches include 1. regular MFCC extraction, 2. MFCC features enhanced by a binary classifier network, and 3. MFCC features improved using a Linear Discriminant Analysis (LDA) process. Finally, the Estimation Algorithm module calculates the glucose levels based on the MFCC inputs. Three regression techniques were implemented: Multi-Layer Perceptron, Support Vector Regression and Regression Tree.

This article presents in Section 2 detailed information regarding the database collection and selection criteria, raw signal processing, feature extraction techniques, and the proposed glucose estimation methods. The performance of each system is reported in Section 3, where the best combination of feature extraction and estimation methods was evaluated using the Clarke Error Grid Analysis and the Park Error Grid. Finally, Section 4 offers final remarks, discusses the implications of the results, and outlines future research directions.

## 2. Materials and Methods

### 2.1. Database Description

The database was collected from 217 patients (57.99% female, 42.01% male) at José Carrasco Arteaga Hospital in Cuenca, Ecuador [13]. The research protocol was approved by the Institutional Review Board of the Universidad San Francisco de Quito, under approval code 2018-017E. Fasting diabetic and non-diabetic patients between 18 and 65 years of age were recruited through a cross-sectional, non-random selection process. All participants were informed about the data collection procedures and provided a written informed consent form.

The PPG data acquisition process involved placing the Empatica E4 wristband sensor (Empatica, Milano, Italy) [14] on the non-dominant arm of the participants for approximately four minutes. Subsequently, the researchers recorded measurements of weight and height, along with additional information regarding age, cardiovascular conditions, and medication use. Blood samples were collected by trained medical personnel from the hospital. Glucose levels were measured using both the Roche Cobas 6000 analyzer series equipment (laboratory glucose) and the AccuChek Performa Nano glucometer (glucometer glucose) for self-monitoring. One sample was obtained from each participant, with no follow-up within a three-month period. The recruited population consisted of 59.36% non-diabetic, 32.34% diabetic and 8.22% pre-diabetic participants. Additionally, 2.76% of the participants were pregnant and 15.20% were diagnosed with hypertension. The main characteristics of both the participants and the recorded samples are presented in Table 1 and Table 2, respectively.

### 2.2. Signal Processing

**Figure 2:**
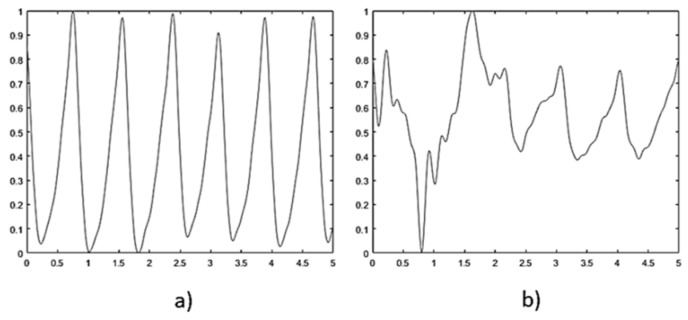
Signal discrimination using Fisher’s Kappa analysis. (**a**) Accepted signal, periodicity index 128.48. (**b**) Discarded signal, periodicity index 43.76.

The Empatica E4 sensor bracelet is a high-precision device, equipped with a PPG sensor operating at a 64 Hz sampling frequency and a sensor output resolution of 0.9 nW per digit. The signal obtained by the sensor is segmented into 5 s fragments for analysis and normalization. Due to user movement interference, the data is not stable across all segments. To discriminate corrupted frames, Fisher’s Kappa analysis for white noise was implemented. This process calculates the ratio between the maximum amplitude of the signal’s periodogram and the meaning of all amplitudes. The analysis provides a metric for signal periodicity, thereby allowing differentiation between PPG fragments with a single periodic component and those affected by noise (see Figure 2).

The procedure discarded about 15% of the blocks as corrupted, obtaining a total of approximately 41 fragments per sample.

### 2.3. Feature Extraction

The database was collected from 217 patients (57.99% female, 42.01% male) at José Carrasco Arteaga Hospital in Cuenca, Ecuador. The research protocol was approved by the Institutional Review Board of the Universidad San Francisco de Quito, under approval code 2018-017E. Fasting diabetic and non-diabetic patients between 18 and 65 years of age were recruited through a cross-sectional, non-random selection process. All participants were informed about the data collection procedures and provided a written informed consent form.

#### 2.3.1. Mel-Frequency Cepstral Coefficients

The extraction of MFCCs is a widely used technique for analyzing signals that contain relevant information in both frequency and cepstral domains. Common applications include speech processing, emotion identification through voice analysis [15] and physiological pattern recognition related to hemodynamic changes [16,17]. Nevertheless, MFCCs have also been employed as a feature extraction technique for inertial signal classification [18], emotion recognition based on EEG signal analysis [19], and cardiac sound classification based on ECG [20,21].

**Figure 3:**
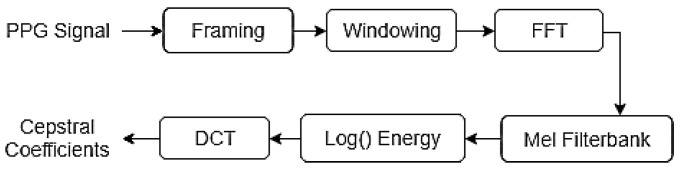
MFCC calculation process.

The MFCC extraction process follows the steps illustrated in Figure 3. The technique analyzes each 5 s PPG fragment by subdividing it into 16 frames of 500 ms, with a 60% overlap. Given that PPG signals are slowly varying over time (with a main frequency around 1.5 Hz), the frame length was set to be considerably longer than that typically used in speech processing applications. A Hamming window is applied during the framing stage to reduce the unwanted effects caused by discontinuities at the frame edges and to emphasize important information at the center of the signal. The spectrum of each frame is then calculated using the Fast Fourier Transform (FFT), which obtains the magnitude distribution across frequencies. For this analysis, only frequencies below 50 Hz were considered relevant for the PPG spectral analysis.

The features related to the source of the signal are contained in the envelope of the power spectrum. To extract these features, a set of triangular filters was applied to the frequency response. The Mel-filterbanks are logarithmically distributed bandpass filters that are multiplied by the spectrum to obtain the logarithmic energy of the signal at each filter. The relationship between the Mel frequency scale and the Hertz frequency scale is shown in Equation (1), where $F_{Mel}$ represents the frequency value on the Mel scale and $F_{Hertz}$ represents the frequency value in Hertz.

(1) $$F_{Mel}=1127ln[1+(F_{Hertz}/700)]$$

The cepstral coefficients are calculated by applying the Discrete Cosine Transform (DCT) to the logarithmic energy of the bandpass filters. DCT is employed because it produces outputs that are decorrelated from one another, concentrating on the relevant signal information in the lower-order coefficients and thus achieving dimensionality reduction. The equation used to compute the DCT is presented in Equation (2), where $E_{n}$ represents the log energy of each filter, N is the number of filters, and m is the number of desired coefficients, which is set to 12 in this case.

(2) $$C_{m}=\sum_{n=1}^{N}Cos\left[k\left(n-0.5\right)\frac{\pi }{N}\;\right]E_{n}\;k=1,2,3\ldots m$$

**Figure 4:**
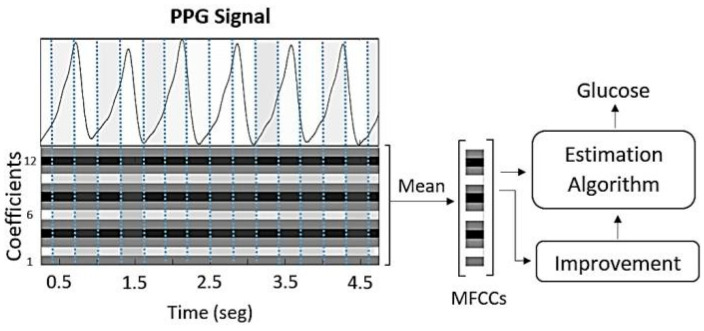
The cepstral coefficients are extracted from the PPG signal and then averaged to serve as inputs for the glucose estimation algorithms.

The logarithmic energy of each frame represents another relevant parameter of the signal and is included as an additional value in the final feature vector. The resulting set of 13 cepstral coefficients captures essential information about both the signal and the physical medium that produced it. To generate a single input vector for each glucose sample used in the estimation algorithms, the mean of all MFCC vectors corresponding to a given glucose value was calculated, resulting in a single 13-coefficient feature vector per sample. Consequently, a complete feature matrix of size 13 × 217 was obtained for the entire database (see Figure 4).

Every frame individually contains characteristics of the vascular system. For the subsequent MFCC improvement techniques, each MFCC frame was paired with its corresponding glucose value to create a new database consisting of 656 feature vectors (16 MFCC frames × 41 signal fragments) for each glucose sample.

#### 2.3.2. MFCCs and Multiplayer Perceptron

Individual PPG signals are similar in both shape and spectrum. The MFCC features of these signals exhibit similarities among each other, making it challenging to establish a relationship that accurately estimates glucose levels for each case. To address this, two MFCC improvement algorithms were implemented to create greater separation between glucose classes and enhance the system performance.

A Multilayer Perceptron (MLP) binary classifier is a learning algorithm capable of predicting the probability of an output state (true/false, 0/1) given a set of inputs. This approach relies on training the network to differentiate MFCC vectors associated with a specific glucose value from those corresponding to other glucose samples. The MFCC vectors of a particular glucose sample were labeled as class 1, while a randomly selected subset of the vectors from the remaining glucose samples was labeled as class 0.

Given the distribution of glucose values in the database, most of the class 0 MFCC vectors statistically represent different glucose values than those of class 1. The MLP network processes the 13 MFCC features as inputs, passing them through two hidden layers of 13 and 10 neurons, respectively, to compute an estimated binary output. This process is repeated for each glucose sample in the database. The logistic sigmoid function was selected as the network activation function due to its capability to model output state probabilities, with the decision boundary set at a threshold of 0.5.

The metrics used to evaluate the system’s classification performance were accuracy and precision. Accuracy measures the proportion of correctly classified samples relative to the total number of samples (Equation (3)), while precision considers only the samples correctly classified as class 1 out of all those classified as class 1 (Equation (4))

(3) $$accuracy=\frac{TP+TN}{n}$$

(4) $$precision=\frac{TP}{TP+FP}$$

where *TP* represents the true positives (class 1), *FP* are the false positives, *TN* represents the true negatives (class 0), and n is the total classified data. Once the network is trained and achieves an acceptable performance in both metrics (over 0.7), the resulting weights of the 10-neuron hidden layer are extracted to perform as a new feature vector for the glucose value. This way, a database of 10 features for 217 glucose samples was obtained by the MFCC-MLP improvement system.

#### 2.3.3. MFCCs and Linear Discriminant Analysis

The second improvement algorithm implemented was Linear Discriminant Analysis (LDA). LDA is a supervised statistical learning technique used to enhance the separation between classes, in this case glucose values, in a lower-dimensional space. The improved MFCC features are obtained by maximizing the between-class variance (or distance) while minimizing the within-class variance or distance ratio between samples that belong to the same class.

The 656 feature vectors corresponding to each glucose value were used as inputs for the calculation of the new lower-dimensional features. To generate a single input vector for each glucose sample to be used in the estimation algorithms, the mean of all the improved vectors associated with a glucose value was calculated, resulting in a single feature vector of 10 coefficients. Consequently, a complete feature matrix of size 10 × 217 was obtained for the entire database.

### 2.4. Estimation Algorithms

Three different glucose estimation algorithms were implemented to evaluate the extracted features. These systems are based on regression techniques and were trained using supervised learning. In addition to the PPG characteristic vectors, specific patient information such as height, weight, body-mass index, diabetes diagnosis (coded as yes = 1, prediabetic = 0.5, no = 0), and age was incorporated into the inputs of the estimation algorithms.

#### 2.4.1. Multiplayer Perceptron

Artificial Neural Networks (ANNs) are a well-known technique for pattern classification and automatic learning that can be applied in medical environments to aid in the diagnosis and prediction of variables, serving as an alternative to traditional statistical models [22]. ANNs are capable of learning hidden relationships within the input data to generate the desired output, providing good performance with relatively simple architectures.

A Multilayer Perceptron (MLP) is a type of ANN whose structure is divided into three main components: the input layer, where the feature vectors of the PPG signals enter the network; the hidden layer, where numerous units called neurons are interconnected according to a specific learning pattern; and the output layer, where the result of the adjusted regression function for glucose estimation is produced.

In this study, the network architecture includes two hidden layers consisting of 10 and 5 neurons, respectively. This parameter configuration was determined using a cross-validation method for each of the feature extraction cases to achieve optimal performance. Regarding the activation function, a radial basis function was selected due to its high performance in linear regression tasks and its ability to approximate any function as a linear combination of Gaussian waves. Additionally, the resilient backpropagation optimization algorithm was preferred over the traditional Levenberg–Marquardt method, as the latter consumes excessive computational resources and significantly increases the time required to optimize the network weights.

#### 2.4.2. Support Vector Regression

Support Vector Regression (SVR) is based on the well-known Support Vector Machine method. SVR provides a reliable technique for function generalization given a limited number of training sets. The algorithm computes a linear regression function in a high-dimensional space, where the input data is mapped through a non-linear transformation to estimate the output. The support vectors are defined in Equation (5), where x denotes the input values, y represents the desired output, and w corresponds to the parameters of the regression function.

(5) y−wx−b≤εwx+b−y≤ε

The margin of fitting of the function is defined by the error tolerance parameter ε, as the regression behaves as a convex optimization problem to minimize (6).

(6) $$y=wx+b\;y^{\prime }=w$$

For the purposes of this study, a radial basis function (RBF) kernel was used as the projection method in the hyperplane to generate the regression function, and the parameter ε was set to a value of 10 in order to avoid data overfitting.

#### 2.4.3. Regression Tree

Regression Trees (RT) are a type of non-linear predictive model that is well suited for data mining and for uncovering hidden relationships among uncorrelated data [23]. A regression tree is composed of decision nodes that represent binary paths the input information can follow to obtain a given response. To initiate the tree construction, the algorithm identifies the binary decision that provides the greatest amount of information about the desired output, generating the root node. Subsequent nodes are created by applying the same division technique until a global data model is established. In cases involving a large feature space, it is recommended to partition the data into smaller regions, where a simpler model can be fitted for each partition. The tree architecture is easy to interpret and can provide valuable insights into the most relevant input features and its interactions.

To prevent overfitting, the decision tree was pruned using a cross-validation method and set to maximum of 500 node splits in its structure. The data was divided into 10 folds for training and validation; when a specific tree size did not perform well on the validation set, the algorithm started pruning nodes to improve generalization performance.

## 3. Results and Discussion

The feature extraction methods combined with the estimation algorithms were evaluated using a cross-validation method based on a 10-fold division of the database. The feature vector database was randomly divided into ten blocks; the system uses nine of these blocks for algorithm training, while the remaining block was used for validation. This process was repeated 10 times until all blocks had been evaluated. This validation approach ensures that each glucose sample is used exactly once for validation, thereby helping to prevent system overfitting.

In the initial procedure, all the estimation algorithms were assessed according to their mean absolute error (MAE) and correlation coefficient (R) between the reference laboratory glucose values and the estimated values (see Table 3 and Table 4).

These metrics were selected because they provide effective insights into the predictive and estimation capabilities of the algorithms. MAE (7) measures magnitude of the errors without considering their direction, whereas the correlation coefficient R (8) evaluates the degree of association between the estimated values and the system’s reference values. Notation for the equations can be found below.

(7) $$MAE=\frac{1}{n}\sum_{i=1}^{n}\left|p_{i}-r_{i}\right|$$

(8) $$R=\frac{\sum_{i=1}^{n}\left[(r_{i}-\bar{r})(p_{i}-\bar{p})\right]}{\sqrt{\sum_{i=1}^{n}{\left(r_{i}-\bar{r}\right)}^{2}\sum_{i=1}^{n}{\left(p_{i}-\bar{p}\right)}^{2}}}$$

The best combination of feature extraction and estimation algorithm was achieved using MFCC features improved with MLP-based enhancements and the Regression Tree (RT) algorithm. The MFCC feature extraction method, paired with the Multilayer Perceptron binary classification, provided effective separation among glucose classes. This result is evident from the performance achieved across all tested estimation algorithms. One possible reason why the RT system outperformed the others is its ability to emphasize the parameters most strongly correlated with the desired output.

Additional metrics used for system performance analysis include the mean squared error (MSE) and the root mean squared error (RMSE), as they measure the average of the squares of the losses generated by the estimation errors of the system. The equations of these metrics are provided below:

(9) $$MAE=\frac{1}{n}\sum_{i=1}^{n}\left|p_{i}-r_{i}\right|$$

(10) $$RMSE=\sqrt{\frac{\sum_{i=1}^{n}{\left(p_{i}-r_{i}\right)}^{2}}{n}}$$

where n represents the total number of samples, $p_{i}$ stands for the predicted or estimated value, and $r_{i}$ represents the real glucose for each sample  i. The performance of the MFCC (improved with MLP) and RT system is summarized in the values of Table 5. The values reflect the effective adjustment of the regression function and yield acceptable test results. The large MSE value is attributed to the high penalization of points that deviate significantly from the regression line; the root square of this value represents a better representation of the dispersion of the validation samples.

**Figure 5:**
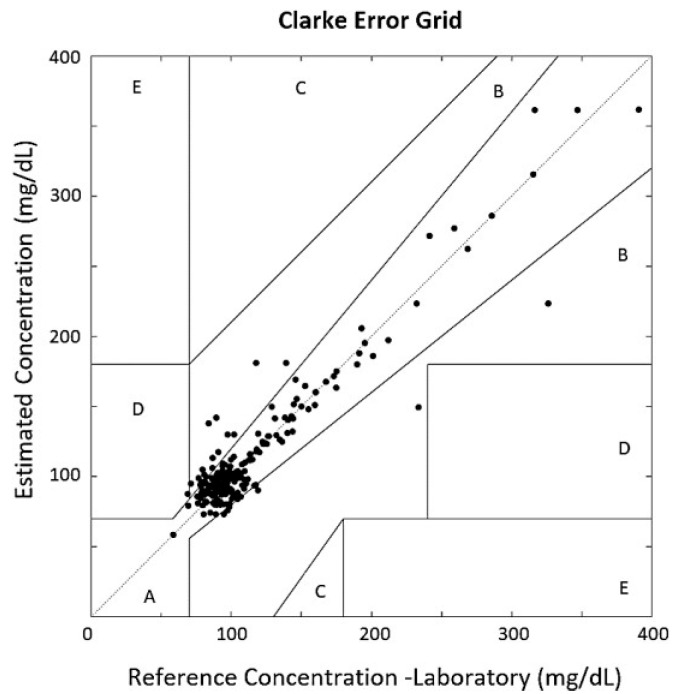
Clarke Error Grid Analysis compares system-estimated glucose values with laboratory references. Zones A,B are clinically acceptable; C–E may lead to inappropriate treatment decisions.

Additionally, the Clarke Error Grid Analysis, Parkes Error Grid and Bland–Altman Plot were used to evaluate system performance. The Clarke Error Grid is subdivided into five zones to visualize data distribution and assess system effectiveness. As shown in Figure 5, 89.86% of the validation values are located in Zone A of the grid, and 9.67% are in Zone B. These zones represent acceptable glucose estimation performance. The remaining 0.47% is located in Zone D, which, along with Zones C and E, indicates poor system performance and is not acceptable for commercial glucose measurement devices.

The glucose values recorded from the hospital laboratory showed a 0.98 correlation when compared with the Accu-Chek glucometer values. Consequently, the estimation results demonstrated similar performance when using glucometer data as reference. In the Clarke Error Grid analysis using the glucometer as the reference, 93.09% of values were found in Zone A, and 6.91% were located in Zone B.

**Figure 6:**
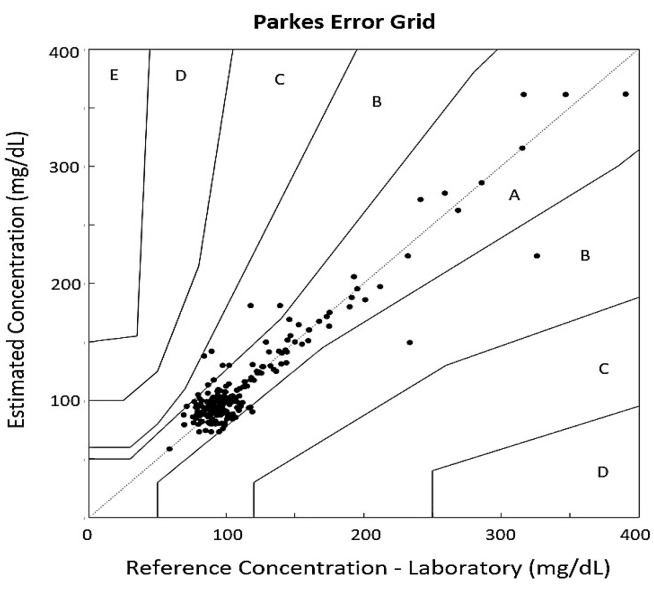
Parkes Error Grid assesses glucose accuracy by comparing model estimates with reference values. Zones A,B are safe states; C–E indicate increasing clinical risk.

**Figure 7:**
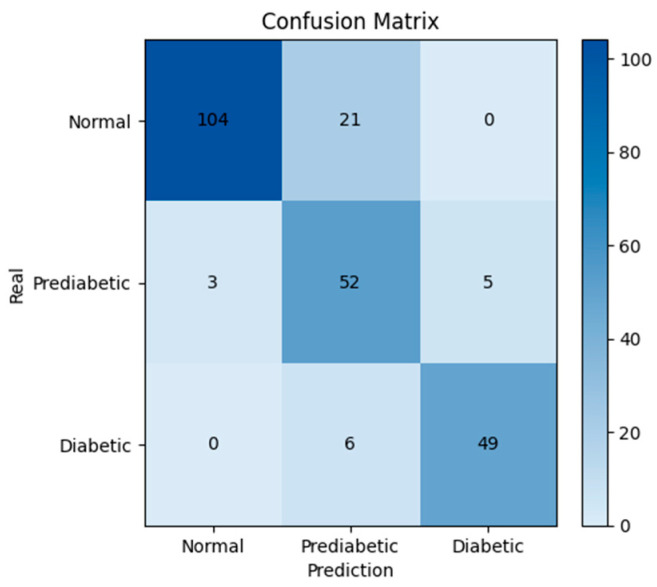
Confusion matrix of the proposed model.

The Parkes Error Grid, also known as the Consensus Grid, is similarly divided into five analysis zones. In this case, 93.54% of the validation values are distributed in Zone A, and 6.45% in Zone B of the grid (see Figure 6). The Bland–Altman plot was used to compare the estimation system with the actual glucose measurement. This plot associates the differences between measures with the averages of the values. The two dotted lines in the graph represent the limits of agreement, where the area between the lines indicates a 95% prediction agreement among the methods (see Figure 7). The plot shows that only 3.68% of the values are located outside those limits, meaning that there are no large discrepancies between the two measuring methods.

The performance results of the proposed system were also compared with those of the other published studies on non-invasive glucose estimation, as summarized in Table 6. As evaluation parameters, the error zones of the Clarke Error Grid were selected for each case, in addition to the total correlation between the estimated and reference sample values.

PPG signal-based systems are emerging methods that have demonstrated effectiveness in glucose monitoring. The proposed system achieves one of the highest correlations between reference and estimated glucose concentration. The algorithm demonstrates strong performance compared to other systems that rely on non-wearable and complex sensors, as well as those requiring pre-calibration for use in some cases. Based on observations using the Clarke Error Grid, it can be noted that the proposed system surpasses most previous studies in terms of accuracy and presents the potential for future use as an alternative to traditional glucose meters.

For the performance evaluation, a confusion matrix based on the comparison between the predicted glucose values and the real reference values was built. To align the evaluation with clinical standards, glucose levels were classified into three relevant categories according to the American Diabetes Association (ADA) guidelines: Normal (<100 mg/dL), Prediabetes (100–125 mg/dL), and Diabetes (≥126 mg/dL). This classification enables the visualization of agreement or disagreement between predicted and reference values, providing a quantitative assessment of the diagnostic accuracy of the proposed system (see Figure 7).

In the confusion matrix, the highest number of correct predictions was observed in the Normal class, with 104 cases correctly classified, and in the Diabetic class, with 49 cases. The Prediabetic class exhibited greater confusion, with 52 correctly identified cases but several misclassifications into the Normal and Diabetic classes. Nevertheless, the model demonstrated a high overall agreement between the predictions and the actual values.

**Figure 8:**
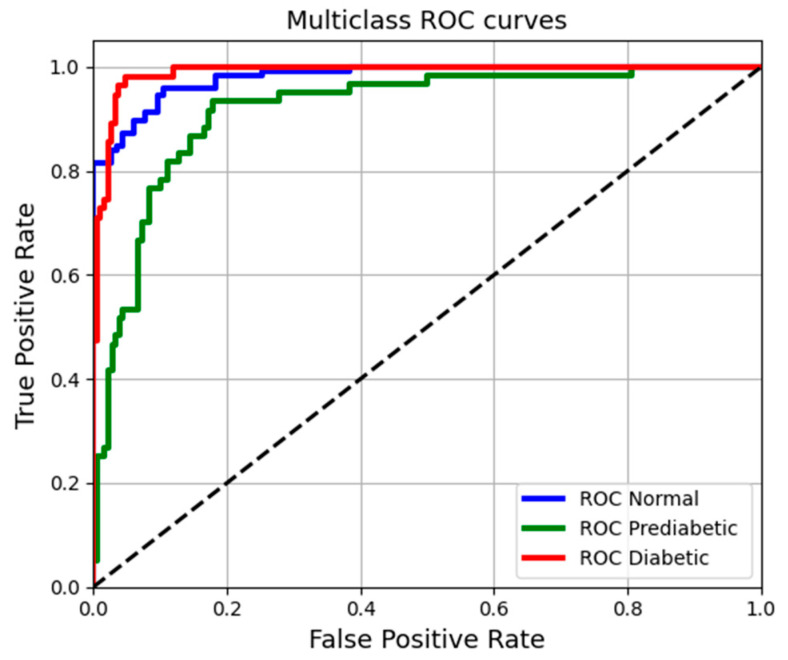
Multiclass ROC curves comparing model performance for Normal, Prediabetic, and Diabetic classifications. Each curve shows the true positive rate versus the false positive rate.

To complement the model evaluation, Receiver Operating Characteristic (ROC) curves were generated for each class (Normal, Prediabetic, and Diabetic), and the area under each curve was calculated (see Figure 8). This method provides insight into the MLP model’s ability to distinguish between the different blood glucose categories.

The figure presents the ROC curves for the three specified classes: Normal, Prediabetes, and Diabetes. The classification demonstrates an outstanding performance, with areas under the curve (AUC) close to one. The curves for the Diabetes (red) and Normal (blue) categories exhibit high true positive rates alongside low false positive rates, indicating strong detection capabilities. In contrast, the Prediabetes curve (green) is less steep, suggesting that the model’s accuracy for identifying prediabetic cases is comparatively lower.

## 4. Conclusions

This article has presented a non-invasive glucose estimation system based on standard PPG signals. The MFCC feature extraction method is computationally simple and does not require prior calibration for its use. To improve signal characterization, two supervised machine learning methods were implemented to maximize the distance between classes and achieve better regression performance. The preferred configuration for the estimation system combined MFCC feature extraction with Multilayer Perceptron binary classification and employed the Regression Tree algorithm for the generation of the estimation function.

The application of speech recognition methods for the characterization of PPG signals has been explored, yielding results that indicate a mean absolute error of 9.85 mg/dL, a root mean squared error of 16.66 mg/dL, and a correlation of 0.94 between the estimated glucose values and the reference values obtained using the Roche Cobas 6000 analyzer series laboratory equipment [29]. The Clarke Error Grid plot revealed that 99.53% of the validation samples were located within Zones A and B of the grid, areas considered acceptable measurements for glucose monitoring equipment. Similarly, all testing samples were distributed in Zones A and B of the Parkes Error Grid, and only 3.68% of the values were placed outside the 95% prediction confidence interval in the Bland–Altman Plot. In future studies, it could be analyzed whether factors such as medication use, insulin administration, and the use of blood thinners introduce bias in glucose estimation based on the extraction of blood viscosity components. Additionally, approaches such as Deep Neural Networks and Heart Rate Variability analysis could further enhance the development of non-invasive glucose measurement systems based on PPG signals.

## Figures and Tables

**Table 1:** Participant physical characteristics.

Measurement	Max	Min.	Mean	Std. Deviation
Age (years)	65	22	48.93	11.27
Weight (kg)	140	38	75.87	16
Height (m)	1.9	1.41	1.6	0.098
$\mathrm{BMI}\;(\mathrm{kg}/m^{2}$)	50.88	17.82	29.41	5.08

**Table 2:** Glucose sample characteristics.

Measurement	Max	Min.	Mean	Std. Deviation
Laboratory Glucose (mg/dL)	390.7	58.6	114.05	50.13
Glucometer Glucose (mg/dL)	363	67	115.66	44.71

**Table 3:** Mean absolute error performance.

Feature Extr\Estimation A.	ANN-MLP	SVR	RT
MFCCs	28.04	25.09	29.84
MFCCs (MLP)	16.38	15.49	9.85
MFCCs (LDA)	15.11	16.86	10.98

**Table 4:** Correlation performance.

Feature Extr\Estimation A.	ANN-MLP	SVR	RT
MFCCs	0.37	0.45	0.33
MFCCs (MLP)	0.76	0.93	0.94
MFCCs (LDA)	0.88	0.79	0.93

**Table 5:** The performance of the MFCCs (improved with MLP) and RT system.

MAE	R	MSE	RMSE
9.85	0.94	277.55	16.66

**Table 6:** Non-invasive glucose system comparison.

Technology	Zone A	Zone B	Other Zones	R
Proposed System	89.86%	9.67%	0.47%	0.94
PPG Signal [24]	89.7%	10.3%	75.87	0.9
PPG Signal [25]	87.7%	10.3%	1.6	0.94
PPG Signal [26]	87.89%	12.11%	-	.
PPG Signal with esp32 [27]	61.88%	38.12%	-	0.8
PPG system Design [28]	92.1%	5.82	-	-

## Data Availability

The data presented in this study are available on request from the first author.
